# Density-dependent feedback and higher-order interactions enable coexistence in phage–bacteria community dynamics

**DOI:** 10.1093/ismejo/wrag041

**Published:** 2026-03-17

**Authors:** Raunak Dey, Ashley R Coenen, Natalie E Solonenko, Marie N Burris, Anna I Mackey, Julia Galasso, Christine L Sun, David Demory, Daniel Muratore, Stephen J Beckett, Matthew B Sullivan, Joshua S Weitz

**Affiliations:** Department of Physics, University of Maryland, College Park, MD 20742, United States; School of Physics, Georgia Institute of Technology, Atlanta, GA 30332, United States; School of Physics, Georgia Institute of Technology, Atlanta, GA 30332, United States; Department of Microbiology, The Ohio State University, Columbus, OH 43210, United States; Center of Microbiome Science, The Ohio State University, Columbus, OH 43210, United States; Department of Microbiology, The Ohio State University, Columbus, OH 43210, United States; Department of Microbiology, The Ohio State University, Columbus, OH 43210, United States; Department of Microbiology, The Ohio State University, Columbus, OH 43210, United States; Department of Microbiology, The Ohio State University, Columbus, OH 43210, United States; Center of Microbiome Science, The Ohio State University, Columbus, OH 43210, United States; School of Biological Sciences, Georgia Institute of Technology, Atlanta, GA 30332, United States; School of Biological Sciences, Georgia Institute of Technology, Atlanta, GA 30332, United States; School of Biological Sciences, Georgia Institute of Technology, Atlanta, GA 30332, United States; Department of Biology, University of Maryland, College Park, MD 20742, United States; University of Maryland Institute for Health Computing, North Bethesda, MD 20852, United States; Department of Microbiology, The Ohio State University, Columbus, OH 43210, United States; Center of Microbiome Science, The Ohio State University, Columbus, OH 43210, United States; Department of Civil, Environmental and Geodetic Engineering, The Ohio State University, Columbus, OH 43210, United States; NSF EMERGE Biology Integration Institute, Ohio State University, Columbus, OH 43210, United States; Department of Physics, University of Maryland, College Park, MD 20742, United States; School of Physics, Georgia Institute of Technology, Atlanta, GA 30332, United States; School of Biological Sciences, Georgia Institute of Technology, Atlanta, GA 30332, United States; Department of Biology, University of Maryland, College Park, MD 20742, United States; University of Maryland Institute for Health Computing, North Bethesda, MD 20852, United States

**Keywords:** community assembly, modeling and ecological theory, temporal dynamics, phage–host interactions, microbial ecology, community ecology, population dynamics, coexistence theory, higher-order interactions

## Abstract

Diverse phage–bacteria communities coexist at high densities in environmental, agricultural, and human-associated microbiomes. Phage–bacteria coexistence is often attributed to coevolutionary processes mediated by complex, pairwise infection networks. Here, using *in vitro* experiments and mathematical models, we explore how higher-order interactions function as a complementary, ecological feedback mechanism to stabilize phage–bacteria communities. To do so, we examine an environmentally derived, synthetic phage–bacteria community comprised of five marine heterotrophic bacteria (*Cellulophaga baltica* and *Pseudoalteromonas* strains) and five associated phage. We used Bayesian inference to reconstruct free phage production in one-step growth experiments and then forecasted pairwise phage–bacteria community dynamics over multiple infection cycles. In contrast to model predictions of rapid bacterial population collapse, each bacterial strain persisted in the community. We hypothesized and then experimentally validated the relevance of infection attenuation at relatively high viral densities. We extended models into a community context, corroborating complex coexistence of all phage and bacteria. Life-history traits inferred in community fits often differed from those inferred in a pairwise context, implicating higher-order interactions as an additional, ecological stabilization mechanism. Follow-up experiments confirm that phage traits (including burst size) can shift when infecting single versus multiple strains. More broadly, these findings suggest that complex community coexistence of phage and bacteria may be more common than anticipated when including feedback mechanisms outside of the growth-dominated regimes of fitted pairwise models that do not reflect the full scope of ecologically relevant contexts.

## 1 Introduction

Bacteriophage (phage) shape bacterial population dynamics in human-associated, built environments, and environmental microbiomes [1–11]. Phage infections of bacteria are highly specific, such that realized interactions between phage and bacteria can be represented as complex, sparse networks [12]. At microevolutionary scales, phage–bacteria coevolve and often generate interaction networks with nested structures such that phage span a range of generalists to specialists and bacteria span a range of permissive to resistant [7]. At larger scales, phage–bacteria interaction networks can be characterized in terms of modules in which a subset of phage preferentially infect restricted subsets of bacteria [8]. Phage–bacteria interaction networks can also exhibit multi-scale structure, e.g. both in large-scale marine surveys [8, 10] and within experimental coevolutionary studies beginning with joint inoculation of a single phage and bacterial type [13]. However, it remains an open challenge to connect pairwise interaction network structure with ecological feedback mechanisms that enable the emergence and maintenance of complex phage–bacteria communities.

Characterizing pairwise interactions is a first step in developing predictive models of community dynamics. For virus–microbe interactions, doing so requires measurements of associated life-history traits. Baseline interactions between phage and bacteria can be characterized in a pairwise fashion given cultured isolates [7, 13, 14]. Ecologically relevant life-history traits include bacterial growth rates and viral adsorption rates, latent periods, and burst sizes [15–17]. Scaling up pairwise interactions to community interactions has been used to predict coarse-grained patterns and processes of microbial community assemblages [18–21]. However, pairwise interactions often fail to capture the emergent stability and coexistence of communities that arise from factors such as higher-order interactions [22–25], context-dependent emergent effects [26–28], temporal partitioning [29], density-dependent feedback [30], and environmental feedback [31]. Pairwise models typically assume static interactions, ignoring the effects of changing environmental conditions and feedback loops [32–34]. These mechanisms may also be relevant in reconciling gaps between predicted community outcomes derived from pairwise interactions between phage and bacteria.

In this paper, we combine ecological theory, models, and direct experimental measurements to assess the limits to predictability of dynamics within a community of intermediate complexity given five bacterial strains from two different species of marine heterotrophic bacteria (*Cellulophaga baltica* (CBA) and *Pseudoalteromonas* (PSA) strains) and five virulent bacteriophage [35–37]. All five bacterial strains and five phage strains were originally isolated from similar conditions in marine waters and coexist in a synthetic community in laboratory conditions. Beginning with pairwise interactions, we used a nonlinear population model to infer life-history traits from short, time-series measurements of phage and bacteria abundance. The nonlinear model predicts rapid bacterial collapse within a few lytic cycles—in direct contrast to experimental evidence from longer time series. Through a process of model-data integration, we find that higher-order interactions and density-dependent feedback can enable phage–bacteria coexistence in complex communities. This phage–bacteria coexistence was not anticipated from models parameterized based on short-term, pairwise experiments in regimes where viral densities are relatively low. As we show, this iterative use of models and experiments reveals mechanisms that may enable coexistence of complex phage and bacterial communities—and provides a caution for extrapolating community-scale outcomes from a narrower scope of conditions than expected in ecologically relevant contexts.

## 2 Materials and methods

### 2.1 Strains and growth conditions

Five bacterial strains were used: CBA strains NN016038, #4 (1), and #18 (2) were isolated from the Baltic Sea in 1994 (NN016038, hereafter CBA 38) and (3) in 2000 (#4 and #18, hereafter CBA 4 and CBA 18 [38]); and PSA sp.H100 (4) and 13–15 (5) were isolated from the North Sea in 1990 (hereafter PSA H100 and PSA 13–15 [39]). Five bacteriophages were used: (i) $\phi$38:1, a podovirus isolated from the Baltic Sea in 2005 on CBA 38, (ii) $\phi$18:2, a siphovirus isolated from the Baltic Sea in 2000 on CBA 18, (iii) $\phi$18:3, a podovirus isolated from the Baltic Sea in 2005 on CBA 18 [38], (iv) PSA-HP1, a podovirus isolated from the North Sea in 1990 on PSA H100, and (v) PSA-HS6, a siphovirus isolated from the North Sea in 1990 on PSA 11–68 (strain not included in this study [40]). Bacteria were grown on *Pseudoalteromonas–Cellulophaga* (PC) plates (20.5 g Sigma Sea Salts, 1.5 g peptone, 1.5 g proteose peptone, 0.6 g yeast extract, 13 g agar/l) at room temperature (RT). Single colonies were inoculated and grown stationary at RT overnight in PC liquid growth medium (20.5 g Sigma Sea Salts, 0.75 g peptone, 0.5 g yeast extract, 0.25 g proteose peptone, 0.25 g casamino acids, 1.5 ml glycerol/l). Phage strains were stored in phage buffer (20.5 g Sigma Sea Salts/l) and plaque forming units (PFUs) enumerated using the agar overlay method [41] with 3.5 ml molten soft agar (20.5 g Sigma Sea Salts and 6 g low melting point agarose/l) and 300 $\mu$l overnight bacterial culture per plate.

### 2.2 Community experiment

The community experiment was performed in triplicate with all five bacterial strains and all five phage strains together. Bacterial strains were inoculated and transferred individually; transfer cultures were pelleted at 4000 g for 10 min and $4\times 10^{8}$ cells of each strain ($2\times 10^{9}$ total cells) were added to 200 ml PC growth medium in each of 6$\times$ 2 l flasks. Phage strains were then added at a multiplicity of infection (MOI) of 0.1 ($4\times 10^{7}$ each phage, $2\times 10^{8}$ total phage) to three flasks; three contained no phage, and samples were taken every 35 min for 15 h 45 min. At each time point, we sampled for OD$_{600}$, CFUs, intracellular qPCR quantification (qINT), and extracellular qPCR quantification (qEXT).

Primers for qPCR were designed to amplify 75–150 bp portions of each bacterial strain or phage, with negligible amplification from other members of the community. The primers were designed using the complete genomes of each bacterial strain or phage. All bacterial and phage genomes are sequenced and publicly available, but 2 of the bacterial genomes were not complete (CBA 4 and PSA sp. H100) and existed in genome fragments. To rectify this, we applied long-read sequencing to resequence and complete those two genomes. In addition, we also resequenced PSA sp. 13–15 because primer design was difficult for the two PSA bacterial strains as they are 99.9% identical genomically.

Primer pairs were tested for efficiency and mis-priming, and we used only primers with >85% efficiency and <10 copies/$\mu$l amplification from other community members. DNA was extracted from qINT samples using the DNeasy Blood and Tissue kit (Qiagen) following the manufacturer’s instructions; qEXT samples were used as-is. qPCR was performed on an Eco Real-Time PCR System (Illumina) with PerfeCTa SYBR Green FastMix Reaction Mix (QuantaBio) in 13 $\mu$l reactions. Per reaction, we used 6.5 $\mu$l PerfeCTa master mix, 0.39 $\mu$l 10 mM forward primer, 0.39 $\mu$l 10 mM reverse primer (Supplementary Information Table S7), 4.72 $\mu$l nuclease-free water, and 1 $\mu$l template. For qINT samples, extracted DNA was used as template, and for qEXT, the 0.2 $\mu$m filtrate was used. Reactions were performed in technical duplicates with a standard curve consisting of 5$\times$ 10-fold dilution series of known concentration of the target strain or phage, used to calculate target sequence copies/$\mu$l. Cycling conditions were as follows: polymerase activation for 5 min at 95$^\circ$C; 40 cycles of 20 s at 95$^\circ$C, 10 s at primer annealing temperature (Supplementary Information Table S7), and 20 s at 72$^\circ$C, and a 55$^\circ$C—95$^\circ$C melt curve.

### 2.3 Single bacteria and pairwise phage–bacteria experiments

Bacterial growth curves to determine doubling times were performed in triplicate in PC growth medium on cultures transferred 1:20 from overnight cultures into new media, grown stationary at RT. A regression of OD$_{600}$ and colony-forming units (CFUs) was constructed using exponential phase time points to estimate cell density from OD readings.

Adsorption of phage to bacterial strains was characterized in triplicate by combining one phage with one bacterial strain and enumerating free (0.2 $\mu$m filtered to remove bacteria) and total phage over time. Pairs with a known interaction were combined at an MOI of 0.1 ($10^{7}$ phage and $10^{8}$ cells/ml), and total and free phage PFUs were enumerated every 3–5 min for 24–25 min to calculate the adsorption constant. Pairs with no known interaction were combined at an MOI of 3 ($3\times 10^{8}$ phage and $10^{8}$ cells/ml), and total and free phage PFUs were enumerated at time intervals for 3 h to examine evidence in support of a statistically significant decrease in free phage. All infections were performed with strains in mid-exponential phase.

One-step growth curves were performed in triplicate to determine phage burst sizes and latent periods. Each phage–bacteria pair was combined at an MOI of 0.1 ($10^{7}$ phage/ml and $10^{8}$ cells/ml) and incubated for 15 min to allow phage to adsorb to cells. The infection was then diluted 1:100 in PC growth medium to reduce the chances of new adsorptions. Total and free phage PFUs were enumerated at regular intervals for 2–4 h. The conventional latent period was determined as the length of time before a significant increase in phage concentration. The average burst size is given by $\langle \beta \rangle = \frac{\langle V_{2}\rangle - \langle V_{1}\rangle }{\langle T_{1}\rangle - \langle V_{1}\rangle }$ where $V$ is the free phage density before ($V_{1}$) and after ($V_{2}$) the burst event, and $T_{1}$ is the total phage density before the burst event. The averages $\langle \cdot \rangle$ are taken over multiple time points and experiment replicates. All infections were performed with strains in mid-exponential phase.

### 2.4 Pairwise experiment for multiple infection cycles

Each bacterial strain was inoculated, transferred, and pelleted as previously described. All pairwise phage–host interactions were tested in triplicate, with triplicate uninfected controls for each strain. Each strain was diluted to $2\times 10^{6}$ CFU/ml in PC media, and a single phage was added at an MOI of 0.1. From this infection, we immediately sampled to quantify total and free phage as previously described, and for intracellular (qINT) and extracellular (qEXT) qPCR. For qPCR, 1 ml was pelleted at 10 000 g for 5 min at 4$^\circ$C. The supernatant was 0.2 $\mu$m filtered to remove any unpelleted bacteria and stored at 4$^\circ$C for qEXT analysis and the pellet was flash frozen in liquid nitrogen and stored at $-80^\circ$C for qINT analysis. We also aliquoted the infections into wells of a 96-well plate and read OD$_{600}$ at 35-min intervals for 15.75 h. At the end of this time, we repeated total phage, free phage, qEXT, and qINT sampling from the infection wells, pelleting 150 $\mu$l instead of 1 ml. DNA was extracted from qINT pellets using the DNeasy Blood and Tissue kit (Qiagen), following the manufacturer’s instructions for Gram-negative bacteria. qPCR was performed to quantify intracellular and extracellular phage and bacterial DNA as previously described at 0, 3, and 15.75 h.

### 2.5 Modeling

We developed a coupled system of nonlinear ordinary differential equations (ODEs) to represent the dynamics of susceptible ($S$), exposed ($E$), and infected ($I$) bacterial cells and viruses ($V$). This Susceptible-Exposed-Infected-Virus (SEIV) model is parameterized by the viral latent period ($\tau$), burst size ($\beta$), adsorption rate ($\phi$), and bacterial growth rates ($r$). We utilized a non-stiff ODE solver with an adaptive time step [42] and fixed constraints. Variation in the latent period distribution is incorporated through a compartmental model of $E$ classes [17] that generates Erlang distributed latent periods [43]. Supplementary Information Section S1.1 contains a full model description. The community SEIV model is scaled up to include interaction specific traits of all nine phage–bacteria pairs. The SEIVD model also includes a state variable representing cellular debris ($D$), a proxy for the cumulative number of lysed cells. We assume debris inhibits infection via a Hill function given by $\frac{1}{1+(D/D_{c_{i}})^{2}}$ given the half-saturation constant $D_{c_{i}}$. Supplementary Information Sections S1.3 and S1.4 provides details on the community SEIV and SEIVD models, respectively.

### 2.6 Inference

We used a delayed rejection adaptive Markov Chain Monte Carlo algorithm to fit the models to the data (including replicates when available) using Bayesian inference [44, 45]. For the one-step growth experiments, the priors were derived from the conventional estimates of the life-history traits. The priors and likelihood functions were chosen to be either truncated normal or log-normal distributions. For the community experiments over multiple infection cycles, the traits of the one-step experiment fail the prior predictive check. A coordinate gradient descent algorithm was used to fit one of the replicates [46], which was used to inform priors, and the rest of the replicates were used to inform the likelihood in the Bayesian inference scheme. In all cases, posterior predictive checks and convergence tests were performed. Supplementary Information Section S1.6 provides a detailed description on inference methods.

## 3 Results

### 3.1 Multiple bacteria and their associated phage coexist in the same community

**Figure 1.**
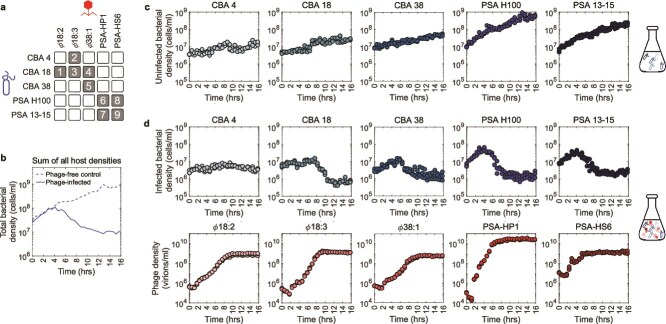
Community experimental data. (a) Adsorption assay experiments reveal an infection matrix with nine out of twenty-five possible infections occur among the strains, each numbered in a shaded gray box. Empty white boxes denote the absence of evidence for infection of the phage strain (in the columns) with the bacterial strain (in the rows). (b) Comparison of total bacterial population dynamics measured in triplicate via qPCR for the phage-free community control (five host strains) and the phage-infected community (five host and five phage strains). (c) Population dynamics of each host from the phage-free community control, where all the bacterial strains are grown together. (d) Population dynamics of each host and phage from the phage-infected community experiment. Five phage and five bacterial strains coexist together in the same flask. All three experimental replicates are shown for each panel. See Supplementary Information Fig. S1 for means and standard deviations of the population dynamics.

We sought to assess whether a complex community of five bacteria that includes three CBA and two PSA isolated from similar environmental conditions could coexist at timescales spanning multiple life cycles when mixed with five associated virulent phage (see Materials and methods for strain details). To do so, we first identified the host range of each of the five phage—identifying nine positive interactions among twenty-five combinations. The resulting infection matrix is modular such that the phage strains associated with either CBA or PSA do not cross-infect the other species (Fig. 1a). Next, we combined all five bacteria at the same initial densities and their five associated phage strains at a fixed MOI of $0.1$. We sampled the *in vitro* community dynamics through triplicate experiments, recording cell and virion densities through intracellular quantitative Polymerase Chain Reaction (qPCR-INT) and extracellular quantitative Polymerase Chain Reaction (qPCR-EXT), respectively, every 35 min for 15.75 h, resulting in 28 time points for each flask (see Materials and methods for further details on culture conditions and sampling methods). All five bacteria and five phage strains coexist for the duration of the experiment and the control (means and standard deviations shown in Supplementary Information Fig. S1 and replicates shown in Fig. 1d). The high-resolution sampling of the phage–bacteria community also revealed subtle transient dynamics. Initially, phage populations decline given adsorption to cells even as cell populations increase given the relatively low initial impact of viral lysis. Subsequently, viral populations increase via infection and lysis (Fig. 1d, bottom row), leading to bacterial density declines $\sim$4–8 h after inoculation that subsequently stabilize to levels between $10^{5}$ cells/ml and $10^{7}$ cells/ml at the conclusion of the experiment. Similarly, phage populations stabilize between $10^{9}$ virions/ml and $10^{11}$ virions/ml, coexisting with, rather than eliminating, the bacterial populations. In contrast, a phage-free control experiment that otherwise followed the same protocols led to the coexistence of all five bacterial strains at densities between $10^{7}$ cells/ml and $10^{9}$ cells/ml (Fig. 1c). The total bacterial population in the phage-free community reached a value $\sim 10^{9}$ cells/ml than that with phage (Fig. 1b). We interpret these data as suggesting that the cumulative impacts of phage infection and lysis, and not resource scarcity, are limiting bacteria populations in the phage-treated community relative to that of the phage-free controls.

### 3.2 Inferring pairwise phage–bacteria dynamics and traits

**Figure 2.**
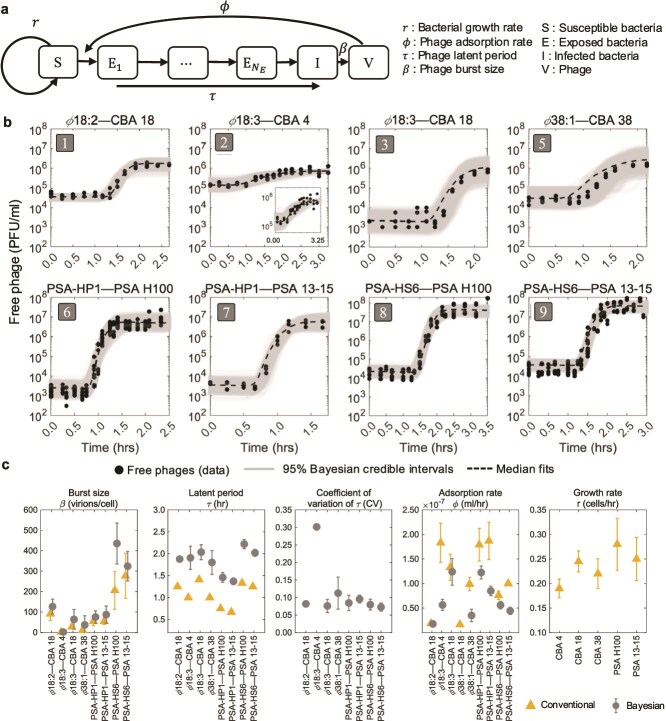
Pairwise interactions between phage and bacteria. (a) Pairwise phage–bacteria dynamics fit with a nonlinear ODE model (described in Supplementary Information S1.1) with a variable number of compartmental stages of infection $N_{E}$ (Supplementary Information Fig. S2), parameterized by the specific phage–bacteria pairwise life-history traits: $r,\phi ,\tau ,\beta$. (b) Free phage density measured through one-step growth curves (black dots) for eight out of nine interacting phage–bacteria pairs. Using the model in (a), the pairwise life-history parameters $\beta$, $\tau$, and $\phi$ are inferred by a Bayesian algorithm. The Bayesian fits are shown in gray lines along with 95% credible interval shaded for each of them. (c) The conventionally inferred life-history traits from single-strain experiments (yellow triangles) such as one-step growth curves, pairwise adsorption assays, and bacterial growth doubling time experiments are compared with Bayesian inferred life-history traits (gray circles) from one-step growth curves; standard deviations shown as error bars. The coefficient of variation for the latent period distribution (related to the inferred number of compartments $N_{E}$ as $\frac{1}{\sqrt{N_{E}+1}}$) is also inferred for each pair. Full details on the fitting statistics are provided in Supplementary Information Figs S9 and S10.

In order to reconcile the finding of elevated phage densities with community coexistence, we set out to quantify bacterial growth rates as well as intracellular and extracellular phage life-history traits and then embed those into pairwise population models (Fig. 2a). We measured the growth rate $r$ through doubling time experiments, finding that CBA strains divided every 4.2–5.8 h and PSA strains divided every 3.6–3.8 h (Fig. 2c). Next, we estimated the adsorption rates of each of the five phage to their respective hosts using adsorption assays with ranges between $\sim 2\times 10^{-8}$ and $2\times 10^{-7}$ ml/h (Fig. 2c). New phage began to appear 0.5 h–1.5 h later. For each interacting phage–bacteria pair, we used a Bayesian MCMC approach to fit an SEIV infection population model to one-step growth curves (Supplementary Information Fig. S2 and Material and Methods S1.1, with prior distributions informed from conventional trait estimates). One-step growth experiments were not obtained for the interaction of phage $\phi$38:1 on CBA 18 due to methodical challenges associated with inefficient infections for this pair [47]. The SEIV model recapitulated phage production dynamics in one-step growth curves (Fig. 2b). Fitted models provide posterior estimates of life-history traits including the adsorption rate $\phi$, mean latent period $\tau$, coefficient of variation of the latent period, CV of $\tau$, and burst size $\beta$. We compared Bayesian inferences of traits with “conventional” methods [15] that utilize the time of first appearance of viruses and viral plateau to infer traits in Fig. 2c (as summarized in Supplementary Information Table S4). The conventional approach to inferring the mean latent period from one-step growth curves systematically underestimates the mean latent period by neglecting variability in latent period outcomes among infected cells in a population [17]. We estimate the coefficient of variation of the latent period to be $\approx$ 0.1 for most strains. As is shown in Fig. 2b, the nonlinear SEIV model of phage–bacteria interactions with inferred life-history traits can recapitulate virus production dynamics over a single lytic period.

### 3.3 Dynamics of pairwise infections over multiple infection cycles

**Figure 3.**
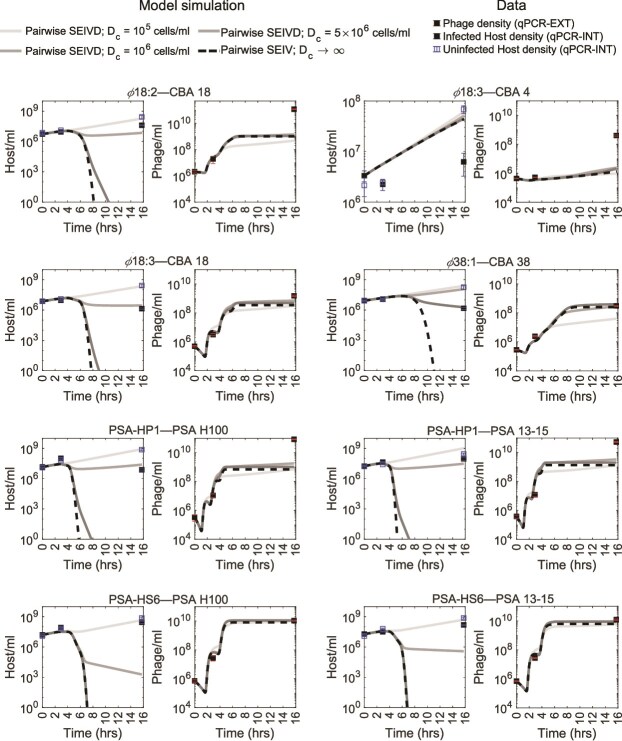
Host densities from pairwise infection experiments over multiple life cycles. For eight phage–host infection pairs, phage and host densities obtained via triplicate qPCR-INT (intracellular) and qPCR-EXT (extracellular) measurements, respectively, for 15.75 h (representing multiple life cycles) are plotted in solid squares. Phage-free control for hosts are plotted in open squares. Pairwise SEIV model (dashed lines; where $D_{c} \rightarrow \infty$ and there is no debris-mediated attenuation of infection) and SEIVD model (solid lines) parameterized through one-step experiments (Fig. 2) and experimentally recorded initial densities are plotted for each experimental pair. Pairwise SEIV model predicts host elimination in seven out of the eight cases in these pairwise experiments, whereas pairwise SEIVD model with infection attenuation qualitatively demonstrates phage–host coexistence for experimental timescales. For the ratios of final to initial host densities, please refer to Supplementary Information Fig. S3.

As a step towards understanding the basis for coexistence at the community scale, we set out to understand if phage–bacteria pairs could persist together over multiple infection cycles. We reinitiated all eight viable, pairwise infection experiments focusing on phage–bacteria pairs for which we have robust parameter estimates (as described in the prior section). In each case, we measured host densities via qPCR at $t=0$, $t=3.5$ h (the longest endpoint within the trait inference experiments) and $t=15.75$ h (consistent with time scales in the community experiment; see Materials and methods). The ratio of final to initial host density in control (phage-free) populations varied from 10 to nearly 100, consistent with host proliferation in the absence of phage (Fig. 3). We hypothesized that addition of phage would lead to the collapse of bacterial populations. Instead, host populations persisted in all pairwise infection cases to the end of the experiment. In all cases, the ratio of final to initial host density in infected populations was lower than that in control populations (see Fig. 3 and Supplementary Information Fig. S3 for further details).

We simulated pairwise SEIV models over multiple infection cycles to see if we could recapitulate the pairwise experimental findings, assuming life-history traits inferred via single-cycle pairwise experiments, as shown in the prior section. The pairwise SEIV models predicted the experimentally observed densities up to 3 h, but then failed to predict densities over longer time scales. Instead of persistence, the pairwise SEIV models predict population crashes within 16 h for seven out of the eight host phage pairs (dashed lines in Fig. 3) and predict substantial host population declines of at least a factor of $10^{2}$ in all cases, except for CBA 4 interacting with $\phi 18:3$. In contrast, we found that five out of eight experiments with infected populations exhibited increased host densities and never exhibited host population declines greater than a factor of $10^{2}$. This finding implies that host growth remains largely in balance and potentially exceeds viral lysis in the majority of pairs, in strong contrast to simulated dynamics in models that inferred apparently efficient lysis within one-step growth experiments.

We hypothesized that the pairwise SEIV models neglected a density-dependent feedback that was not observed when fitting to growth-dominated early time data. As such, we extended the SEIV model to include the impact of a negative feedback mechanism in which bacterial lysis inhibits new infections, e.g. potentially due to inhibitory effects of cellular debris on viral adsorption and lysis. Rather than representing a confirmed mechanistic pathway, the SEIVD model is an effective description of cellular-level mechanisms that could impede phage infection centered around one phenomenological feature: infection attenuation increases nonlinearly as a function of lysed cells. This new pairwise SEIVD model (where ‘D’ represents debris, a proxy for lysed cells, see Supplementary Information Model S1.2) includes a negative feedback loop in which the strength of infection attenuation is given by a Hill function tuned by a critical concentration, $D_{c}$. We choose a Hill coefficient of 2 for all cases. Given $D_{c}$ values ranging from $10^{5}$ to $5\times 10^{6}$ cells/ml, the model predicts bacterial persistence with minimal impacts on phage population dynamics. This finding suggests that additional stabilizing mechanisms can be present even in pairwise contexts at high densities that may also be relevant when analyzing community dynamics.

### 3.4 Scaling-up pairwise infection models do not recapitulate community level dynamics

**Figure 4.**
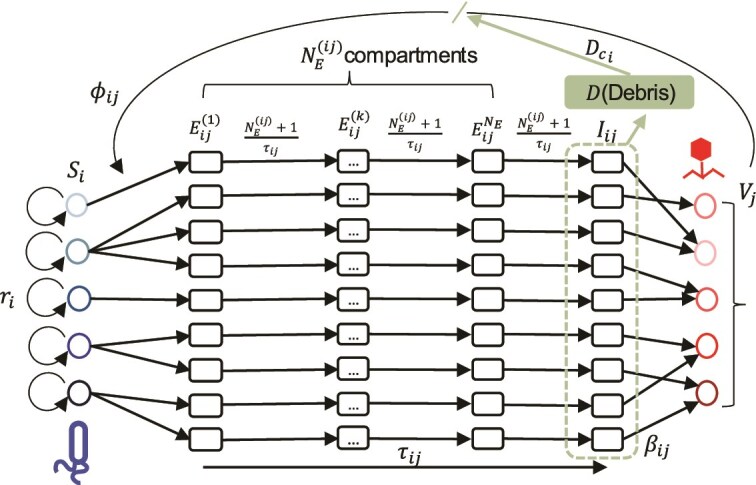
SEIV- and SEIVD-based scaled-up community model. Using the infection matrix in Fig. 1b the model is scaled up to include the nine interactions between five bacterial and five phage strains and respective number of exposed classes $N_{E}^{(ij)}$ for each interaction. $S_{i}$, $E_{ij}$, and $I_{ij}$ are, respectively, susceptible, exposed, and infected stages in the sequential infection for each bacteria $i$ by phage $j$. This SEIV community model is parameterized by the phage infection traits of the constituent pairs of the community, such as latent periods ($\tau _{ij}$), adsorption rates ($\phi _{ij}$), burst sizes ($\beta _{ij}$) and bacterial growth rates ($r_{i}$). For the SEIVD model, we include a state variable $D$, which represents the dead cell density that can attenuate new infections, given a critical debris concentration $D_{c_{i}}$. See main text and Supplementary Information S1.3 and S1.4 for the full description of the SEIV and SEIVD models, respectively.

We scaled up the pairwise SEIV and SEIVD models to a community context by incorporating cross-infections as obtained through adsorption assay experiments (Fig. 1a). In a community model, each susceptible bacteria population, $i$, with density $S_{i}$ can be infected by each of its associated phage, $j$, with density $V_{j}$. For each of the nine pairs, we account for latent period heterogeneity by modulating the number of classes of “exposed” compartments, $E_{ij}$. We specifically excluded the potential for multiple infections of a single cell, either by the same or different phage. The community models include the potential to re-estimate life-history traits and a strain-specific critical debris concentration, $D_{c_{i}}$ (Fig. 4). Complete details of the community SEIV and SEIVD models are described in Supplementary Information S1.3 and S1.4.

**Figure 5.**
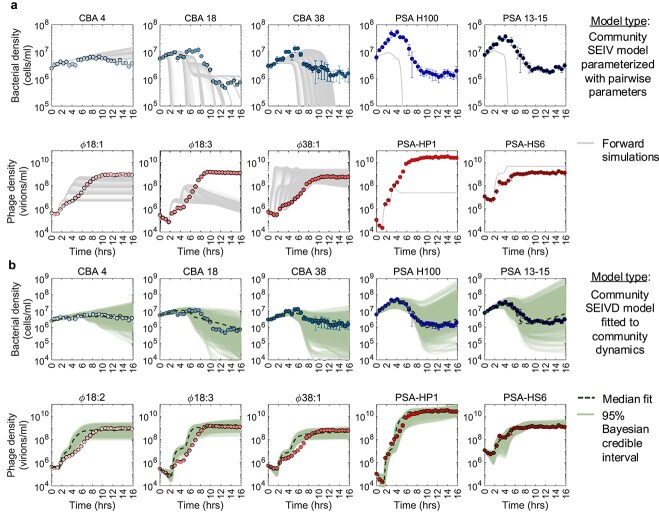
Comparison of observed versus simulated phage–bacteria population dynamics. The mean of the population density time series for all the five bacterial hosts and five phage obtained from triplicate experiments are shown (circles), with their respective standard deviations. (a) The life-history traits inferred from single strain experiments and one-step growth curves in Fig. 2 are directly used to parameterize the scaled-up SEIV community model, where uncertainty in model outcome (forward simulations) is associated with uncertainty regarding the burst size and latent period of $\phi$38:1 on CBA 18 derived from literature (parameters summarized in Supplementary Information Table S4). (b) The community SEIVD model is used to infer community-level life-history traits. Simulations from 95% Bayesian credible intervals of the posterior distribution are shown, with median fits indicated in dashed lines. Priors and posterior distributions are detailed in Supplementary Information Fig. S12. Additional information on fits is available in Supplementary Information Fig. S13 for the MCMC chains and Supplementary Information Figs S14 and S15 for the MCMC convergence statistics.

We simulated the community SEIV model using the pairwise Bayesian median posterior estimates of the viral burst sizes, adsorption rates, average latent periods and their variability for the eight inferred interactions, and bacterial growth rates (Fig. 2c and Supplementary Information Table S4, and Figs S9 and S10). As noted, one-step phage growth parameters could not be inferred for the infection of $\phi$38:1 on CBA 18. Instead, we used a range of parameter values derived from the literature [47]. The community SEIV model parameterized by pairwise-fitted infection traits failed to capture the observed community dynamics. As in the pairwise simulations over multiple infection cycles (Fig. 3), the community SEIV model predicts that all bacterial populations should decrease rapidly given the explosive growth of phage populations (Fig. 5a). Even when accounting for variability in life-history traits, the models predict decreases exceeding two orders of magnitude within 10 h post-exposure to viruses. This bacterial population collapse (not observed in experiments and akin to that shown in pairwise SEIV models) arises via rapid increases and then stabilization of viral populations.

### 3.5 Integrating higher-order interactions and density-dependent feedback in the community model enables complex coexistence

We hypothesize that density-dependent feedback can stabilize complex phage–host communities. To evaluate this hypothesis, we simulated the SEIVD community model parameterized by pairwise parameters and a broad range of independently sampled $D_{c_{i}}$ values utilized in simulating the pairwise experiments. We observed that the community model with density-dependent infection attenuation prevents extinction of host strains but fails to quantitatively recapitulate the phage or host densities (Supplementary InformationFig. S4a). One potential explanation is that life-history traits of phage might differ in community versus pairwise contexts. To evaluate this hypothesis, we refit the community SEIV model to the first 6.4 h of community dynamics, intentionally excluding density-dependent infection attenuation (see Supplementary Information Section S1.6.4). The re-fit community SEIV model quantitatively captured the population dynamics of all phage strains for the entire 15.75 h experiment (Supplementary Information Fig. S4b, bottom) while only capturing bacterial dynamics for the first $\sim$ 6 h (Supplementary Information Fig. S4b, top). Nevertheless, the improvement of community SEIV model fits on the shorter timescale suggests that viral life-history traits differ between pairwise and community contexts (Supplementary Information Table S9).

Finally, we combined both putative stabilizing mechanisms together in a single model: refitting the SEIVD model by performing MCMC-based Bayesian inference on the triplicate population time-series data to estimate the joint posterior distribution of the phage–bacteria life-history traits and $D_{c_{i}}$ values. This SEIVD community model can quantitatively fit both the experimentally recorded bacterial and phage time-series data, unlike either the scaled-up pairwise model with density-dependent infection attenuation or the re-fit SEIV community model (Fig. 5b, additional robustness analysis in Supplementary Information Figs S5 and S6, parameter summary and statistics in Supplementary Information Table S5 and Figs S12–S15). The model-predicted phage dynamics include both initial decreases associated with virion adsorption and subsequent production that eventually stabilizes at levels consistent with observations. Likewise, the ensemble of bacterial density dynamics includes stabilization in all cases, as well as the potential for bacterial population rebounds even in the absence of phage-resistance.

### 3.6 Experimentally testing putative higher-order interaction mechanisms

**Figure 6.**
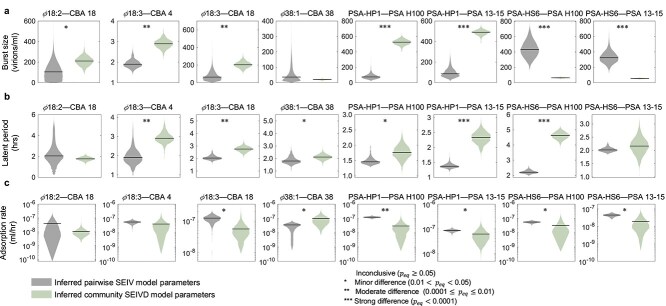
Violin plots for pairwise versus community phage traits. Phage traits inferred via Bayesian methods with pairwise SEIV and community SEIVD models (corresponding to the fitted dynamics in Fig. 2b and Fig. 5b, respectively) are compared with violin plots for the eight phage–host interactions described in Fig. 2. In each panel, the left and right violin plots denote inferred pairwise SEIV and community SEIVD model parameters, respectively. Subfigures (a), (b), and (c) show burst size, latent period, and adsorption rate, respectively. We compared the traits using a Bayesian test for equivalence [48] and the posterior probability of practical equivalence, $p_{\textrm{eq}}$, is defined through a prespecified region of equivalence (analogous to $P$ value). Materials and methods are outlined in Supplementary Information section S1.7, see Supplementary Information Table S6 for detailed statistics.

We set out to test both of the hypothesized mechanisms that shape community-scale coexistence in the SEIVD model: higher order interactions and density-dependent infection attenuation. First, we compared viral traits inferred from pairwise experiments and community experiments inferred via the pairwise SEIV and community SEIVD. We found moderate to strong shifts in traits for 11 trait parameters, whereas 8 of them showed minor differences, and 5 were inconclusive (Fig. 6 and Supplementary Information Table S6 for statistical comparison). The SEIVD community model predicted strong shifts in burst sizes, e.g. the community estimated burst size of PSA-HP1 is larger and that of PSA-HS6 is smaller compared with values inferred in pairwise experiments. To test these unexpected shifts, we performed one-step growth experiments of the PSA strains with all hosts combined (one phage with the five host subcommunity). We found that in the subcommunity of PSA hosts the burst size of PSA-HP1 increases, whereas the burst size of PSA-HS6 decreases relative to the pairwise case, consistent with model-inferred predictions (Supplementary Information S2.3, Fig. S7).

In order to test for density-dependent attenuation of infection, we added bacteria-filtered and phage-inactivated spent media from 12 h of the community experiment to fresh pairwise infections for all nine phage–bacteria infection pairs. In each case, we contrasted dynamics with that of including the host alone and the phage–bacteria infection pair without community spent media. Host densities remained elevated for all strains in the phage-free control. For 3 CBA infections and 1 PSA infection, namely, $\phi 18:3$ on CBA 4, $\phi 18:3$ on CBA 18, $\phi 38:1$ on CBA 38, and PSA-HP1 on PSA 13–15, the host densities fall below the limit of detection in the infection-only control (without community lysate). In contrast, the host persists when community spent media is added along with the phage (Supplementary Information Fig. S8), consistent with a density-dependent attenuation of infection and lysis.

## 4 Discussion

In this work, we explored mechanisms of coexistence within a synthetic marine phage–bacteria community. In contrast to model predictions derived from short-term pairwise phage–bacteria experiments, we found that individual phage–bacteria pairs and complex multi-strain communities can coexist over multiple infection cycles. Through a process of iterative model-data integration, we hypothesized that higher-order interactions stabilize community dynamics. These interactions include context-dependent shifts in life-history traits and infection attenuation at relatively high viral densities. By incorporating both types of higher-order interaction mechanisms, we quantitatively recapitulate community time-series data and the coexistence of all five heterotrophic bacteria and five associated phage over a 15.75 h time series experiment (far longer than the typical infection cycles of 1–2 h). In doing so, we also experimentally validated the model-inferred higher-order interaction mechanisms: (i) higher order interactions such that life-history traits exhibit directional shifts in community versus pairwise experiments, and, (ii) density-dependent attenuation, such that phage lysates diminish the intensity of infection-induced lysis.

Central to our analysis was the development of a nonlinear population dynamics model that incorporated the combined effects of density-dependent infection attenuation and higher order interactions. Both mechanisms were essential to reconcile the potential for rapid lysis when viral densities are low and the coexistence of bacteria with viruses when viral densities are high. First, our finding of density-dependent infection attenuation might be due to accumulation of cellular debris that changes host physiology and/or viral infectiousness [49]. The spent media experiments alone (Supplementary Information Fig. S8) do not necessarily rule out additional inhibitory effects that might occur via feedback in a growing bacterial community. This finding also suggests caution is warranted in extrapolating from short- to long-term dynamics. Ecological models that are fit to dynamics in experimentally convenient conditions may not yet fully represent feedback across biologically relevant regimes. Second, higher-order interactions are increasingly considered to be potentially powerful drivers of coexistence [19, 50, 51], including in microbial communities [24, 25, 52–56]. Our work reinforces the ecological relevance of earlier findings that life-history traits can also depend on context [26, 57], e.g. phage latent period may decrease in response to lower resources [58] and phage growth can be affected by bacterial growth rate [59].

Despite being able to quantitatively recapitulate complex community dynamics, our modeling and inference framework comes with caveats. A salient feature of our ecological models is the explicit integration of the variability in the latent period observed in prior phage host systems [60–63] modeled via multi-compartment stages of infection progression [17, 64]. For simplicity, we assumed that the variability in this latent period is the same for all pairwise interactions in the community SEIVD model. In reality, we expect variability to be strain dependent. Another key assumption is that bacterial traits are constant in time, whereas long-term coexistence would need to account for nuanced bacteria–bacteria interactions as well. Furthermore, we have assumed that each bacterial strain is infected by at most one virus. Previous studies have shown that the outcomes of infection may differ depending on the multiplicity of infection [65–67] and/or communication systems [68]. Additionally, in the community and the pairwise SEIVD models, infection attenuation only becomes relevant at later stages of the experiments. Multiple biochemical pathways that might be responsible for infection attenuation at timescales of multiple infection cycles or at higher cell densities, including those mediated via debris, is abstracted through this effective mechanism. Finally, we have not included the impacts of phage-resistance or counter-defense amongst phage—nor did we observe the emergence of phage-resistant bacterial strains in our experiments. The repeatability of strain dynamics and the absence of bacterial regrowth even as total densities were 100-fold less than observed in phage-free controls suggests that phage-resistance (even if present) is not ecologically relevant on these time scales in this system. Exploring the interface between resistance and counter-resistance will be critical in understanding longer term, eco-evolutionary dynamics of phage–bacteria communities.

In summary, our work suggests that pairwise interactions inform but do not wholly determine community-level interactions. We find that higher-order interactions matter and that assuming rapid pairwise lysis does not necessarily explain phage–bacteria community dynamics and coexistence. The community models and inference framework developed here may be of service in exploring the generality of these feedback mechanisms in environmental, agricultural, and human-associated microbiomes [69–71]. In doing so, our work also presents a caution: scaling up results from pairwise interaction outcomes to community dynamics will likely require accounting for density-dependent feedback and higher-order interactions. Identifying the relevance of both mechanisms is likely to improve the predictive capacity of community-scale models as well as provide into the coexistence of diverse populations of viruses and bacteria.

## Supplementary Material

wrag041_Supplemental_Files

## Data Availability

The datasets and code used for this project are available at: https://github.com/RaunakDey/VIMIMO_paper and are archived on Zenodo, https://doi.org/10.5281/zenodo.17526176.
